# Supplementary material for: Potential Circulating Biomarkers of Recurrence after Hepatic Resection or Liver Transplantation in Hepatocellular Carcinoma Patients
Source: Cancers (Basel). 2020 May 18;12(5):1275. doi: 10.3390/cancers12051275 (PMC7281651; doi:10.3390/cancers12051275)
Supplement: Supplementary file 1 [file cancers-12-01275-s001.pdf]

Supplementary Figure Legend

Fig. S1. Analysis of downstream effectors of IL-10 pathway using The Cancer Genome Atlas (TCGA) data. A, As expected, expression of *IL10RA, JAK1, IL10RB, JAK1* and *TYK2* clustered in the analyzed tumor samples. **B**, Significant correlation between *IL10RA* and *JAK1* expression in HCC tissues. **C**, Using a threshold of >2 z-score for mRNA expression for both *Il10RA* and *JAK1*, we found that tumors with high levels of Il10RA or JAK1 had a median overall-survival of 37.29 months, compared to control tumors (58.84 months, P = 0.0210, Chi square).

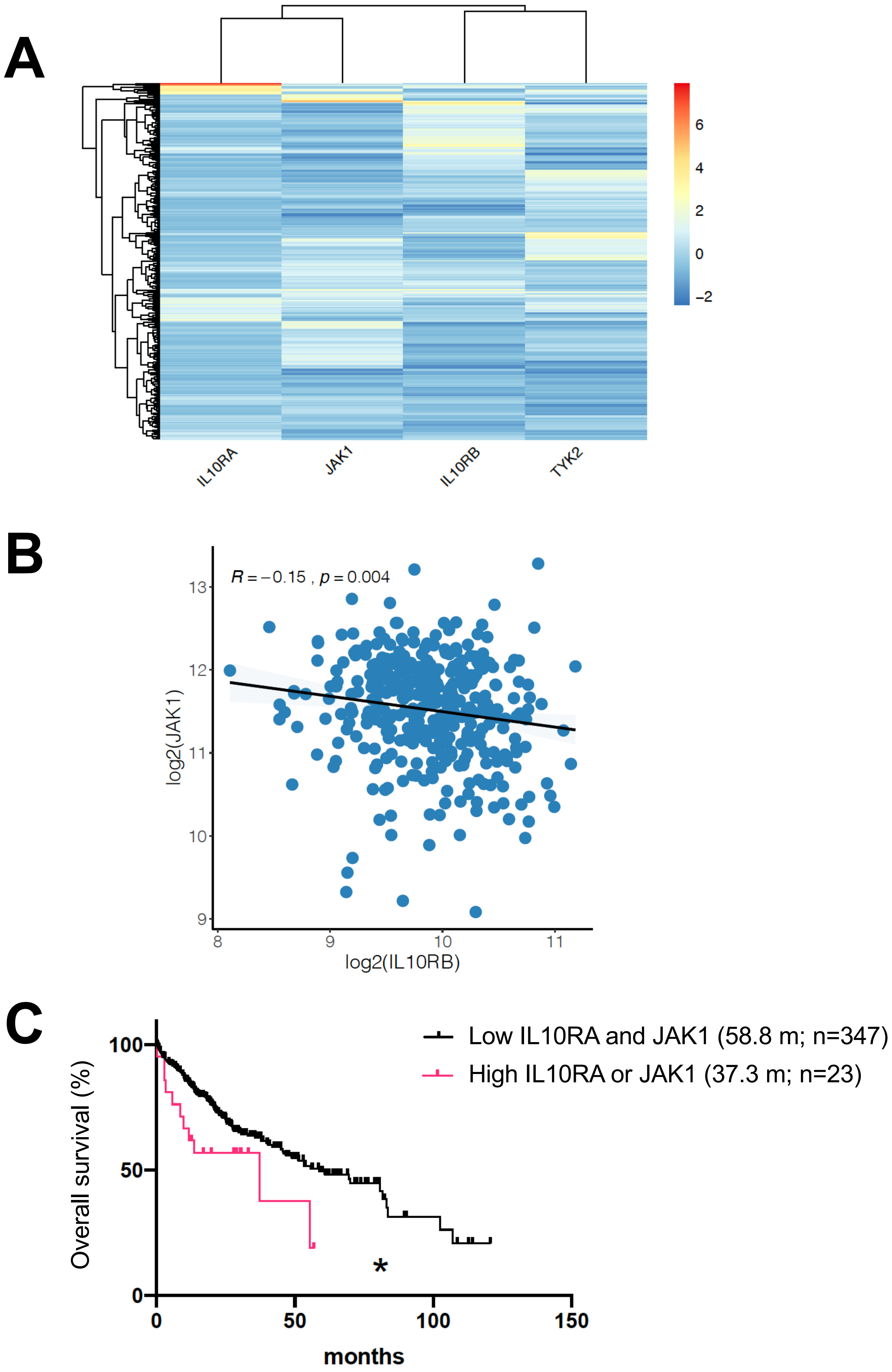
A
6
4
2
0
−2
IL10RA
JAK1
IL10RB
TYK2
B
R = −0.15 , p = 0.004
13
12
11
10
9
log2(JAK1)
8
9
10
11
log2(IL10RB)
C
Low IL10RA and JAK1 (58.8 m; n=347)
High IL10RA or JAK1 (37.3 m; n=23)
100
50
0
Overall survival (%)
*
0
50
100
150
months

Supplementary Table S1: Comparison of preoperative plasma versus serum biomarkers in patients undergoing liver resection. Data are shown as median values and interquartile ranges, with p values from the exact Wilcoxon/exact Fisher test.

Biomarker	Plasma (n)	Serum (n)	p-value
**IFN-γ (pg/ml)**	7.5 [5.6,12.8] (45)	9.2 [5.2,14.3] (69)	0.56
**IL-6 (pg/ml)**	1.60 [1.60,4.31] (45)	2.27 [1.60,5.06] (69)	0.24
**IL-8 (pg/ml)**	12.8 [7.3,18.5] (45)	31.3 [22.1,57.1] (69)	<0.0001
**IL-10 (pg/ml)**	0.77 [0.57,1.99] (45)	0.91 [0.63,1.50] (69)	0.57
**TNF-α (pg/ml)**	3.4 [2.7,4.1] (45)	3.7 [2.7,4.7] (69)	0.43
**bFGF (pg/ml)**	3.8 [2.5,8.5] (45)	6.7 [3.0,19.6] (69)	0.027
**PlGF (pg/ml)**	34.5 [25.3,42.9] (45)	35.9 [29.3,43.1] (69)	0.48
**sVEGFR1-1 (pg/ml)**	115 [86,217] (45)	95 [76,114] (69)	0.013
**sTIE2 (pg/ml)**	5386 [4618,7415] (45)	4602 [3580,5956] (69)	0.0089
**VEGF (pg/ml)**	84 [42,148] (45)	502 [223,1014] (69)	<0.0001
**VEGF-C (pg/ml)**	98 [89,193] (45)	515 [267,747] (69)	<0.0001
**VEGF-D (pg/ml)**	696 [542,986] (45)	1156 [933,1662] (69)	<0.0001
**AFP (ng/ml)**	12.5 [5.6,20.0] (32)	13.5 [3.7,20.0] (69)	0.77

AFP, alphafetoprotein; bFGF, basic fibroblast growth factor; IFN, interferon; IL, interleukin; LR liver resection; LT, liver transplantation; PlGF, placental growth factor; sTIE2, soluble angiopoietin receptor; VEGF, vascular endothelial growth factor; VEGFR, VEGF receptor; TNF, tumor necrosis factor.

Supplementary Table S2. Analysis of clinical parameters and circulating biomarker of interest stratified by Milan criteria in HCC patients undergoing liver transplantation.

Biomarkers	Within Milan Criteria	Outside Milan Criteria	P-value
**Number of deaths**	11/34 pts (32.4%)	5/13 pts (38.5%)	0.74
**Number of deaths or recurrences**	11/34 pts (32.4%)	6/13 pts (46.2%)	0.50
**AFP (>20 ng/ml)**	12/34 pts (35.3%)	2/13 pts (15.4%)	0.29
**AFP truncated at 20 ng/ml**	8.1 [3.5, 20.0] (n=34)	7.8 [3.3, 9.2] (n=13)	0.49
**VEGF (pg/ml)**	80.3 [40.4, 248.6] (n=34)	106.5 [40.0, 210.9] (n=13)	0.95
**VEGF-C (pg/ml)**	138.6 [89.0,180.2] (n=34)	121.1 [89.4, 144.0] (n=13)	0.65

AFP, alphafetoprotein; VEGF, vascular endothelial growth factor.
